# Joint estimation of relaxation and diffusion tissue parameters for prostate cancer with relaxation-VERDICT MRI

**DOI:** 10.1038/s41598-023-30182-1

**Published:** 2023-02-21

**Authors:** Marco Palombo, Vanya Valindria, Saurabh Singh, Eleni Chiou, Francesco Giganti, Hayley Pye, Hayley C. Whitaker, David Atkinson, Shonit Punwani, Daniel C. Alexander, Eleftheria Panagiotaki

**Affiliations:** 1 Centre for Medical Image Computing, Department of Computer Science, University College London, London, UK; 2 Cardiff University Brain Research Imaging Centre, School of Psychology, Cardiff University, Maindy Road, Cardiff, CF24 4HQ UK; 3 School of Computer Science and Informatics, Cardiff University, Cardiff, UK; 4 Centre for Medical Imaging, University College London, London, UK; 5 Division of Surgery and Interventional Science, University College London, London, UK; 6 Department of Radiology, University College London Hospital NHS Foundation Trust, London, UK; 7 Molecular Diagnostics and Therapeutics Group, Division of Surgery & Interventional Science, University College London, London, UK

**Keywords:** Cancer, Magnetic resonance imaging

## Abstract

This work presents a biophysical model of diffusion and relaxation MRI for prostate called relaxation vascular, extracellular and restricted diffusion for cytometry in tumours (rVERDICT). The model includes compartment-specific relaxation effects providing T1/T2 estimates and microstructural parameters unbiased by relaxation properties of the tissue. 44 men with suspected prostate cancer (PCa) underwent multiparametric MRI (mp-MRI) and VERDICT-MRI followed by targeted biopsy. We estimate joint diffusion and relaxation prostate tissue parameters with rVERDICT using deep neural networks for fast fitting. We tested the feasibility of rVERDICT estimates for Gleason grade discrimination and compared with classic VERDICT and the apparent diffusion coefficient (ADC) from mp-MRI. The rVERDICT intracellular volume fraction f_ic_ discriminated between Gleason 3 + 3 and 3 + 4 (*p* = 0.003) and Gleason 3 + 4 and ≥ 4 + 3 (*p* = 0.040), outperforming classic VERDICT and the ADC from mp-MRI. To evaluate the relaxation estimates we compare against independent multi-TE acquisitions, showing that the rVERDICT T2 values are not significantly different from those estimated with the independent multi-TE acquisition (*p* > 0.05). Also, rVERDICT parameters exhibited high repeatability when rescanning five patients (R^2^ = 0.79–0.98; CV = 1–7%; ICC = 92–98%). The rVERDICT model allows for accurate, fast and repeatable estimation of diffusion and relaxation properties of PCa sensitive enough to discriminate Gleason grades 3 + 3, 3 + 4 and ≥ 4 + 3.

## Introduction

As for many cancers, definitive prostate cancer (PCa) diagnosis relies on biopsies^1^. This invasive procedure can have serious side effects, such as infection, bleeding and urinary retention, significantly impacting quality of life^2,3^. Recent advances in medical imaging have played a key role in improving PCa detection. For instance, multi-parametric MRI (mp-MRI), consisting of T2-weighted, diffusion-weighted and dynamic contrast-enhanced imaging sequences has been incorporated into the National Institute for Health and Care Excellence (NICE) guidelines for PCa diagnosis^4^. However, whilst mp-MRI has a 90% sensitivity for detection of significant cancer, it’s specificity is moderate at 50%^5^; resulting in 1 in 2 men still needing to undergo an unnecessary biopsy^2^. Significant cancer is generally defined by the presence of Gleason pattern 4 tumour within a biopsy^6–9^. Reliably identifying lesions on mp-MRI that contain Gleason pattern 4 disease from those with non-significant cancer (Gleason 3 + 3) or no cancer remains an unmet clinical need.

To address this, microstructure imaging techniques based on diffusion-weighted MRI (DW-MRI)^10–12^ offer sensitivity and specificity to microstructure changes well above the simple apparent diffusion coefficient (ADC), conventionally acquired as part of standard mp-MRI protocols^13^. In particular, the Vascular, Extracellular and Restricted Diffusion for Cytometry in Tumours (VERDICT) technique^11,14^ was one of the first showing histological specificity both ex-vivo and in-vivo (clinically and preclinically)^11,14–16^.

The VERDICT model for prostate assumes there are three major tissue compartments that mostly contribute to the measured DW-MRI signal: intra-cellular (ic), intra-vascular (vasc) and extra-cellular/extra-vascular (ees), and these are non-exchanging (i.e. fully impermeable to water). Several studies^11,14,15^ validated these assumptions under the experimental conditions of the optimized DW-MRI acquisition for VERDICT in prostate^17^. Specifically, the VERDICT intracellular signal fraction (f_ic_) correlated with only epithelial cells, while the stroma contribution was captured by the extracellular-extravascular compartment (f_ees_)^15^. These findings were also supported by an in vivo VERDICT validation study^18^, showing very high correlation (r = 0.96, *p* = 0.002) between in vivo VERDICT f_ic_ and epithelial volume fraction from histology. Together with f_ic_ and f_ees_, VERDICT also estimates the MR apparent cell radius (R) and provides a derived measure of cellularity (f_ic_/R^3^).

Results from the recent clinical trial INNOVATE^19^ reveal that VERDICT f_ic_ can discriminate between Gleason 3 + 3 and 3 + 4 lesions (AUC = 0.93, *p* = 0.002)^16^ and that its diagnostic performance in identifying lesions with clinically significant PCa (AUC = 0.96) is higher than that of ADC from mp-MRI (AUC = 0.85, *p* < 0.001) and prostate-specific antigen density (AUC = 0.74, *p* < 0.001)^20^.

However, VERDICT is currently limited in estimating only diffusion parameters without accounting for the inherent relaxation properties of the tissue^21–29^. This leads to potentially uncertain accuracy of microstructural parameters, which could limit their sensitivity. Indeed, relaxometry parameters such as T2 relaxation time have also shown capability to discriminate Gleason grades 3 and 4^21,24,25^. Most importantly, works exploiting joint relaxation-diffusion analysis^29–40^ have shown that these two types of parameters often contain complementary information that can enhance the sensitivity and specificity of non-invasive MRI to pathological tissues.

In this work, we hypothesise that an extended VERDICT model capturing both relaxation and diffusion effects can enhance the accuracy of both types of estimates and improve Gleason grade discrimination. Therefore, we propose a new relaxation-VERDICT (rVERDICT) model that extends the VERDICT model by including compartment-specific relaxation times to estimate jointly the diffusion and relaxation parameters in prostate.

The new rVERDICT model parameterises the T2 relaxation of the intracellular compartment by T2_ic_, and that of vascular and extracellular/extravascular compartments by the same T2_vasc/ees_. It also includes the T1 relaxation contribution from the whole tissue as a single pool. For rVERDICT, the same assumptions as VERDICT for prostate apply, with the additional assumptions about the MR relaxation tissue properties based on currently available experimental evidence^26–28,30,32,33,41–43^. Our choice of a single T1 pool is supported by current literature, showing that it is possible to reliably identify only a single T1 compartment of T1 ~ [1500–3000] ms^27,28^. Kjaer et al.^26^ also acknowledged that a longer T1 compartment likely exists, but lamented the impossibility to measure it within clinical SNR and time-constraints. For the T2 relaxation, we assume the same T2_vasc/ees_ for the vascular and extracellular-extravascular components. This is supported by previous work which showed that in prostate tissue it is possible to reliably distinguish only two compartments with different T2 values: a slow one, with T2 ~ [160–1300] ms and a fast one with T2 ~ [40–100] ms^42^. In rVERDICT we assume that the fast T2 compartment is the intracellular space and the slow T2 compartment the vascular (T2 of oxygenated and deoxygenated blood being ~ 150–250 ms, at 3 T and normal hematocrit level ~ 0.45^43^) and the extracellular-extravascular (typical luminal T2 ~ [160–1300] ms^42^) space. Stroma is not explicitly modelled.

For the estimation of the rVERDICT parameters we use deep neural networks (DNNs) to reduce computational time and enable on-the-fly analysis. This work capitalizes on the VERDICT imaging protocol, which is feasible on clinical scanners, and exploits joint relaxation-diffusion analysis providing a new approach of modelling VERDICT data that harnesses all the information available from the multi-TE DW-MRI VERDICT acquisition.

In this study, we first describe our demographic data and show an example of all the new rVERDICT parameteric maps for a representative participant. Then, we investigate the accuracy, precision and robustness of the DNN estimates of all rVERDICT model parameters using numerical simulations. We assess the repeatability of rVERDICT model parameters estimates using scan-rescan data from the INNOVATE clinical trial; and we validate rVERDICT estimates of prostate T2 relaxation times comparing them with the estimated T2 values from independent multi-TE data from the INNOVATE clinical trial. Finally, we demonstrate the improvements in Gleason grade discrimination provided by the new rVERDICT comparing our results with the classic VERDICT and ADC from mp-MRI of the same 44 participants from the INNOVATE clinical trial with suspected PCa that underwent mp-MRI and VERDICT-MRI followed by targeted biopsy. We discuss the implications of these results for the clinical diagnosis of PCa, compare our findings with current literature and discuss the impact of modelling assumptions, the limitations of this study and future developments.

## Results

### Demographic data

**Figure 1:**
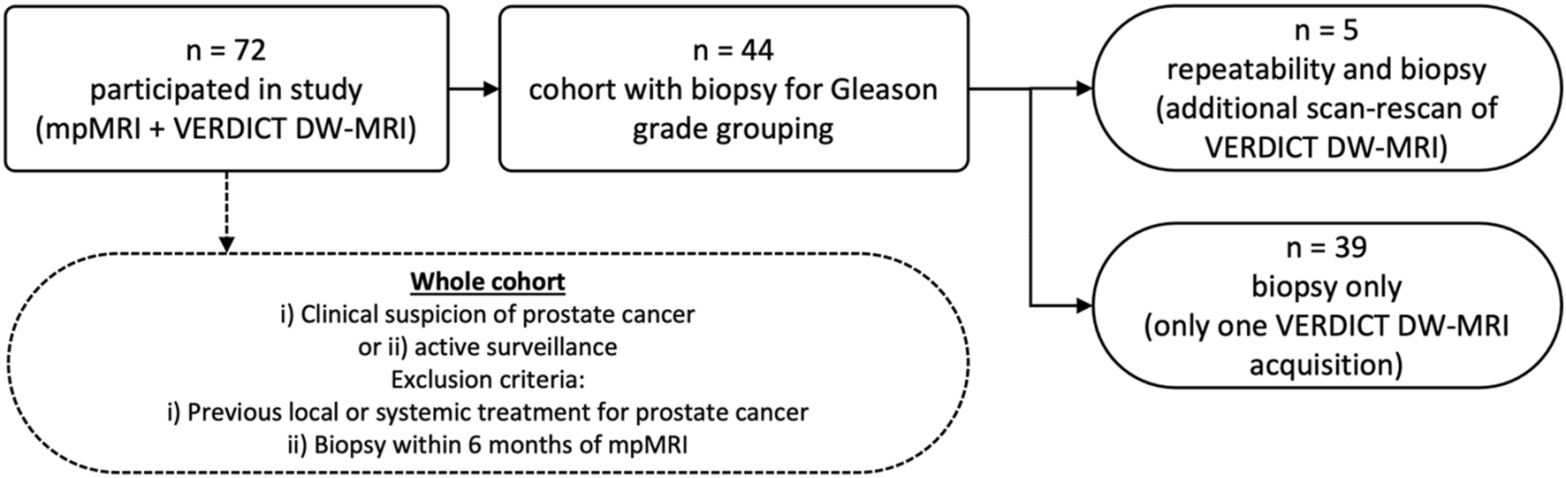
Participation flow diagram. mp-MRI = multiparametric MRI. Among the recruited 44 participants, only five underwent targeted biopsy and were scanned twice for repeatability analysis, while the remaining 39 underwent targeted biopsy only, without scan-rescan.

Figure 1 presents a participation flow diagram. There were 37 cancer lesions in the investigated cohort (n = 44), and 22 regions that were determined as benign tissue on biopsy. Median prostate-specific antigen (PSA) level was 7.0 ng/mL (range = 1.0–71.0 ng/mL), the median time between VERDICT MRI and biopsy was 66.9 days (range = 8–167 days). Of the 37 cancer lesions, 6 were Gleason grade 3 + 3, 18 were 3 + 4, and 13 were ≥ 4 + 3. Table 1 provides a summary of the demographic data.

**Table 1:** Summary of demographic data.

Parameter	Cohort with biopsy
Number of participants	44
Median age (y)	67 (49–79)
Median PSA level (ng/ml)	7.96 (0.83–72.11)
Highest Gleason grade of biopsied index lesion	
Benign	22
3 + 3	6
3 + 4	18
≥ 4 + 3	13
Median no. of total cores	23 (6–36)
Median no. of sites	2 (1–13)
Median no. of positive cores	5 (1–15)
Median maximum cancer core length (mm)	8 (1–14)
Median maximum cancer core length (%)	75 (10–100)
Median Prostate volume (ml)	43 (15–108)

Except where indicated, data are numbers of participants. Numbers in parentheses are ranges.

*PSA* Prostate specific antigen.

### Exemplar rVERDICT parametric maps through DNN

**Figure 2:**
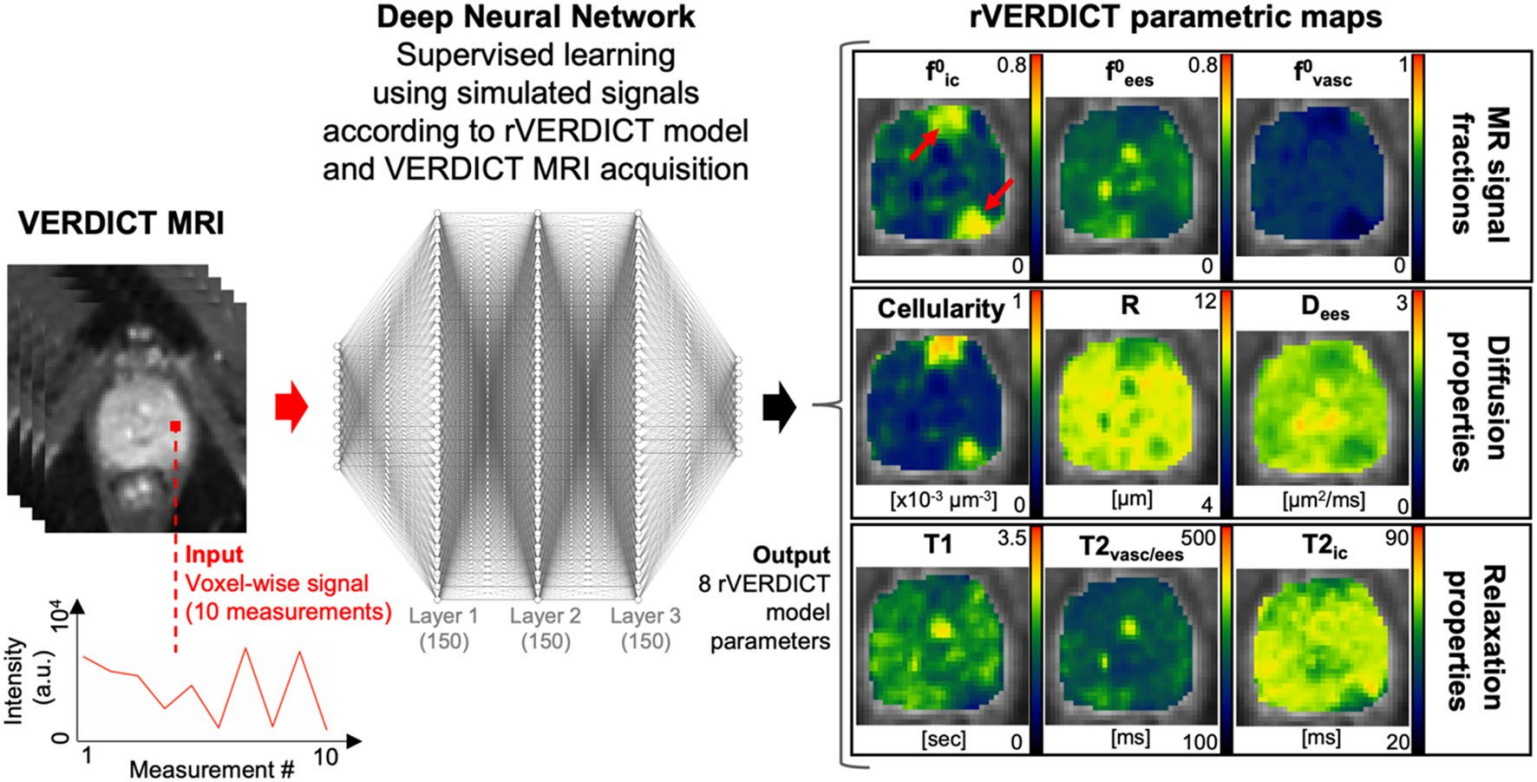
Examples of rVERDICT model parametric maps for a representative participant in our cohort: age late 60’s, PSA 5.21 left posterior lesion Gleason 3 + 4 maximum cancer core length (MCCL) 14 mm, left anterior lesion Gleason 3 + 3. A flowchart of the rVERDICT fitting using a fully connected deep neural network (DNN) is also shown. The DNN takes voxel-wise signals as input and outputs voxel-wise values of the eight rVERDICT model parameters. It is trained in a supervised fashion, using simulated noisy signals according to the rVERDICT model and the VERDICT MRI acquisition. Exemplar training data are shown in Supplementary Fig. S2.

An example of the parametric maps from the new rVERDICT is shown in Fig. 2 for a representative participant from our cohort. In addition to the equivalent parametric maps obtainable with classic VERDICT analysis (i.e. intra-cellular signal fraction f_ic_, extracellular-extravascular signal fraction f_ees_, MR apparent cell radius R and derived MR apparent cellularity), rVERDICT also enables to estimate the MR relaxation properties, such as the apparent intracellular T2 (T2_ic_); the apparent vascular and extracellular-extravascular T2 (T2_vasc/ees_), and the apparent T1 relaxation time. The direct comparison of rVERDICT parametric maps with the classic VERDICT counterparts is shown in the Supplementary Fig. S1. Figure 2 also shows the architecture of the DNN used for the model parameter inference, which is performed voxel-wise after having trained the DNN in a supervised fashion using numerically simulated signals from the rVERDICT model and the same VERDICT MRI acquisition used to collect the real data.

### DNN model fitting performance

**Figure 3:**
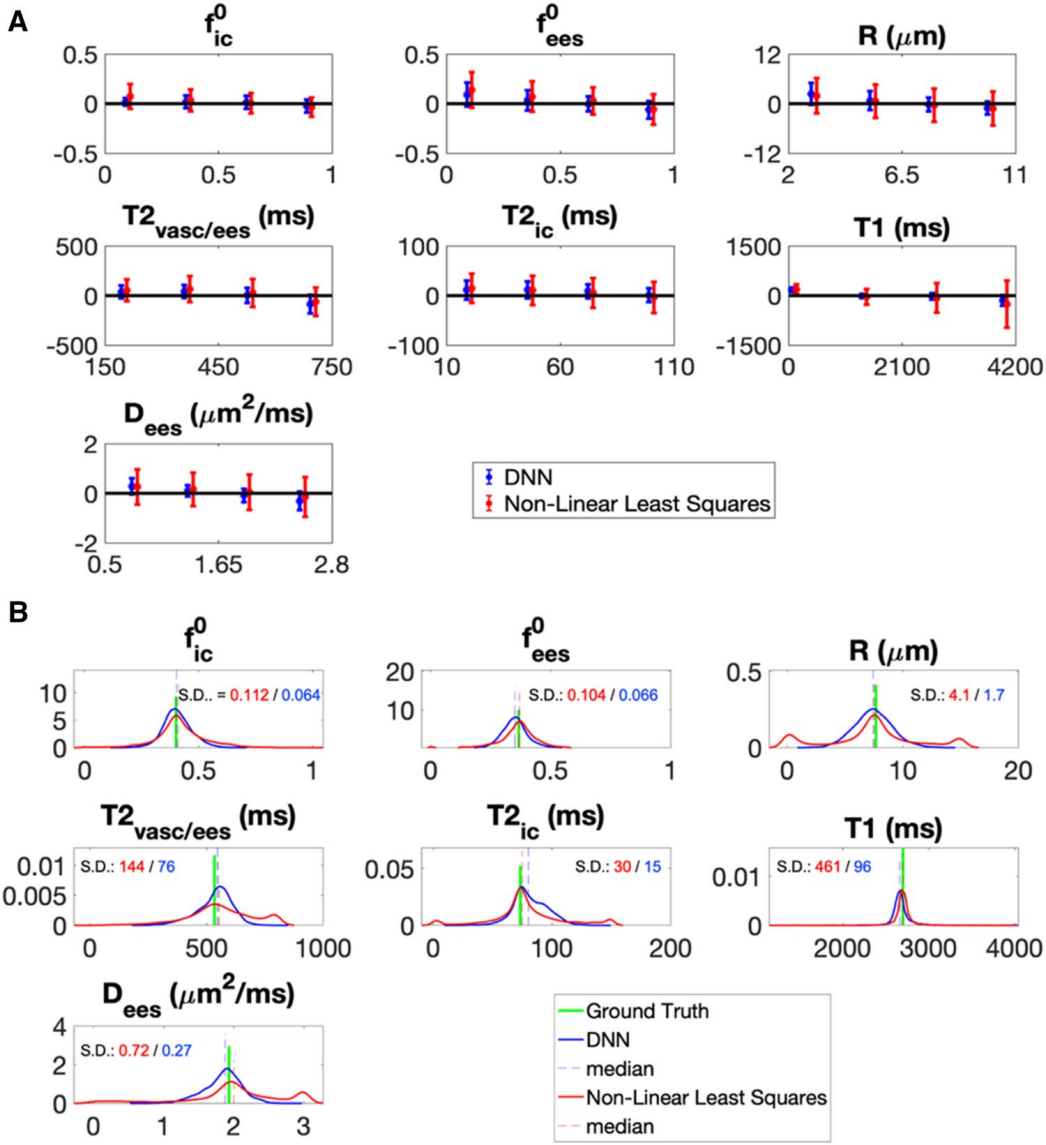
Accuracy and precision of model fitting. (**A**) The mean (data points) and variance (error bars) of the difference between the prediction for DNN or conventional non-linear least squares optimization and the ground truth values are plotted against the known ground truth from numerical simulations. The line at zero difference is also plotted as straight black line to aid appreciating the accuracy of the prediction from both methods (higher the accuracy, closer the mean difference to zero). The variance of the difference (error bars) is a good indicator of the precision of the estimation: smaller the variance, higher the precision. To make the results visually clear, data points for the non-linear least squares were purposely moved slighted to the right. (**B**) The probability density distribution of the estimates of the seven rVERDICT model parameters (S0 was fixed to 1) are plotted for seven ground truth values representative of PCa in TZ and PZ and 4096 different random realizations of the other parameters, for both DNN and conventional non-linear least squares optimisation. The wider the distribution, the less robust the estimation and the lower the precision due to degeneracy and/or spurious minima. We quantified the width of the distributions through their standard deviations (S.D.) reported in each plot with corresponding matching colours.

Numerical simulations show that, for all the rVERDICT parameters, the DNN have similar accuracy (i.e. same bias) but higher precision and robustness to noise and local minima (i.e. lower dispersion) than conventional non-linear least squares minimisation (NLLS): the average bias for DNN is − 13 ± 36% versus − 18 ± 50% for NLLS; the average dispersion for DNN is 34 ± 41% versus 71 ± 108% for NLLS (Fig. 3A). Similar results were obtained when simulating model parameters combinations mirroring values reported for PCa in peripheral zone (PZ) and transition zone (TZ)^32,33,44^ (Fig. 3B). The DNN estimation is also ~ 60 times faster than NLLS.

### Repeatability

**Figure 4:**
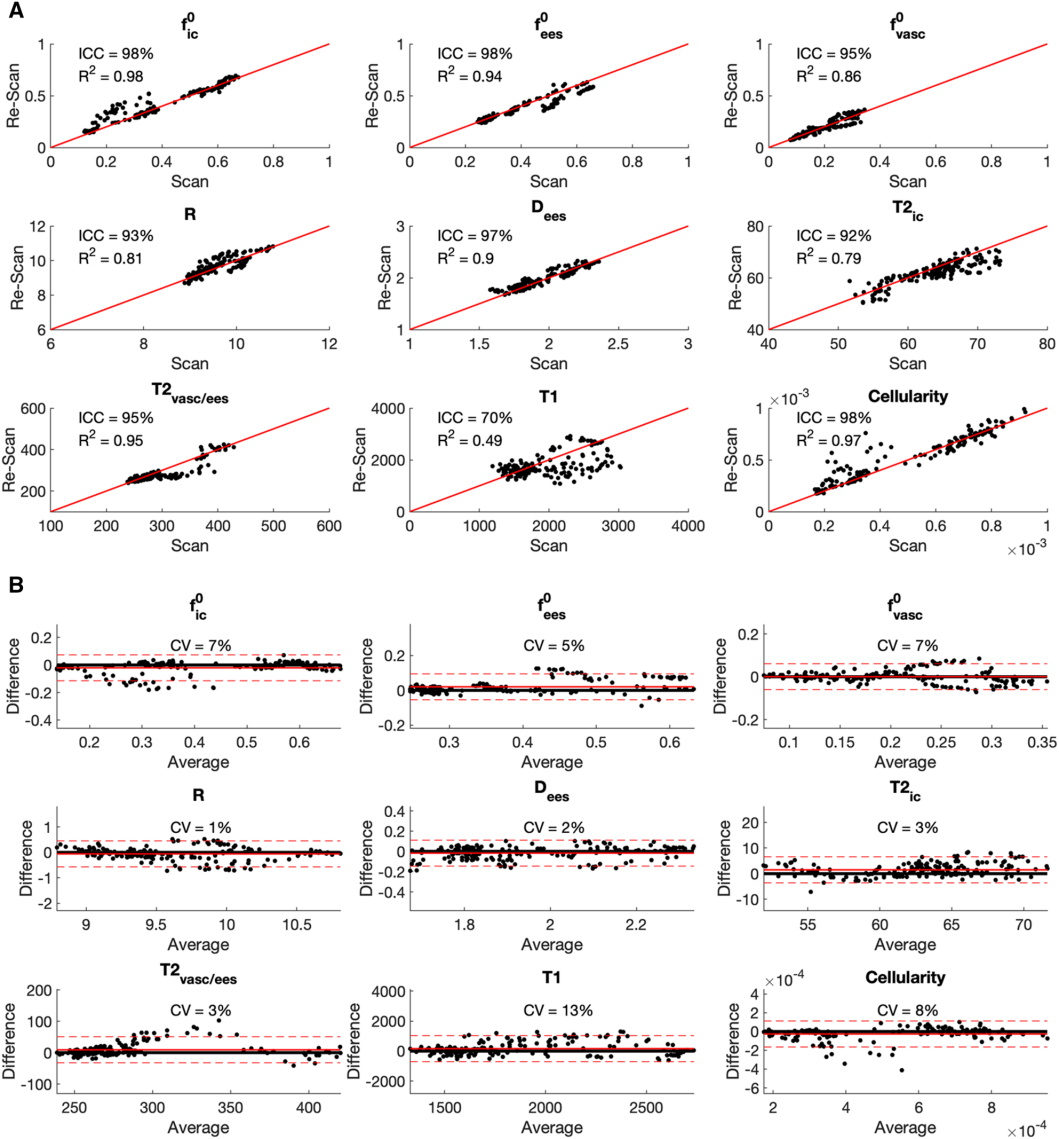
Repeatability of the rVERDICT parameters. (**A**) Correlation plots for all the rVERDICT parameters in the scan/rescan study. The corresponding R^2^ and ICC are reported for each of them, together with the identity line. (**B**) Bland–Altman plots of the scan/rescan estimates for all the rVERDICT parameters. The corresponding CV is reported for each of them, together with the average (straight red line) ± 1.96 standard deviation (dashed red lines) of the difference. The dimensional parameters are in μm (apparent cell radius R); μm^−3^ (Cellularity); μm^2^/ms (extracellular-extravascular apparent diffusivity Dees) and ms (T1, intracellular T2_ic_ and vascular/extracellular-extravascular T2_vasc/ees_).

Region-of-interest (ROI) based repeatability analysis on five scan-rescan datasets show high repeatability of all rVERDICT model parameters estimates. For the diffusion and T2 relaxation parameters from rVERDICT, the adjusted coefficient of determination R^2^ = [0.79–0.98]; the coefficient of variation CV = [1–7%]; and the intraclass correlation coefficient ICC = [92–98%]. The correlation plots and Bland–Altman plots for all the rVERDICT parameters are reported in Fig. 4.

### Comparison of T2 estimates from rVERDICT and T2-relaxometry MRI

**Figure 5:**
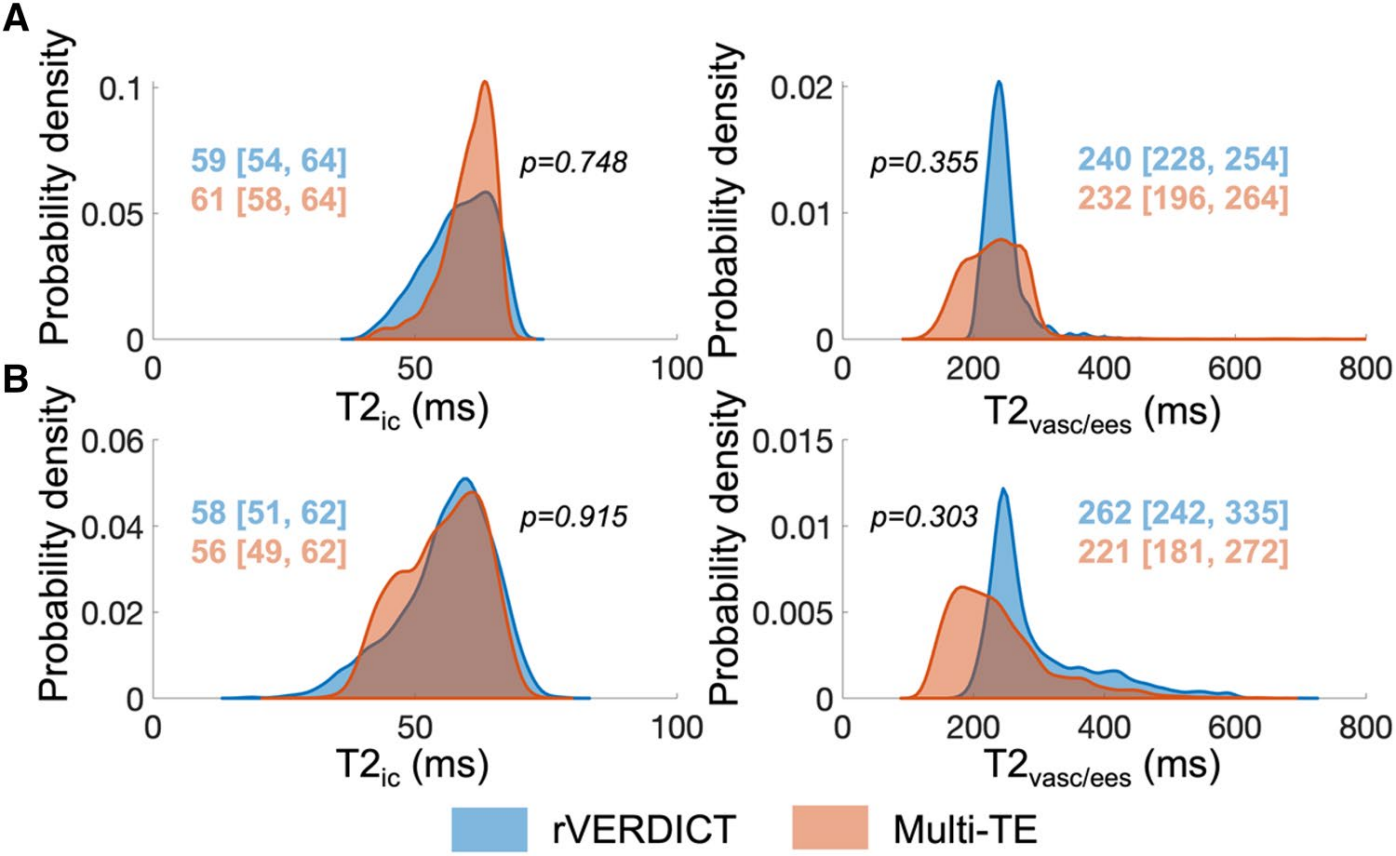
Comparison of T2 estimates from rVERDICT and independent measurements using a multi-TE acquisition. The distributions show the probability density function of the estimated T2 values for all the voxels within the prostate volume for the best (**A**) and worst (**B**) cases. Median and [25th, 75th] percentiles for each distribution are also reported, together with the *p* values from a two-sided Wilcoxon rank sum test.

Comparison of the distributions of T2 values estimated with rVERDICT and independent multi-TE acquisition for the best and worst cases (in terms of comparable median values) in our cohort are reported in Fig. 5. We found median values not statistically different (*p* > 0.05) between the two methods and similar interquartile ranges. The estimated compartmental T2 values are T2_ic_ ~ 60 ms and T2_vasc/ees_ ~ 250 ms; in agreement with current literature^26,27,30,32,33,42,43^.

### Gleason grades discrimination

**Figure 6:**
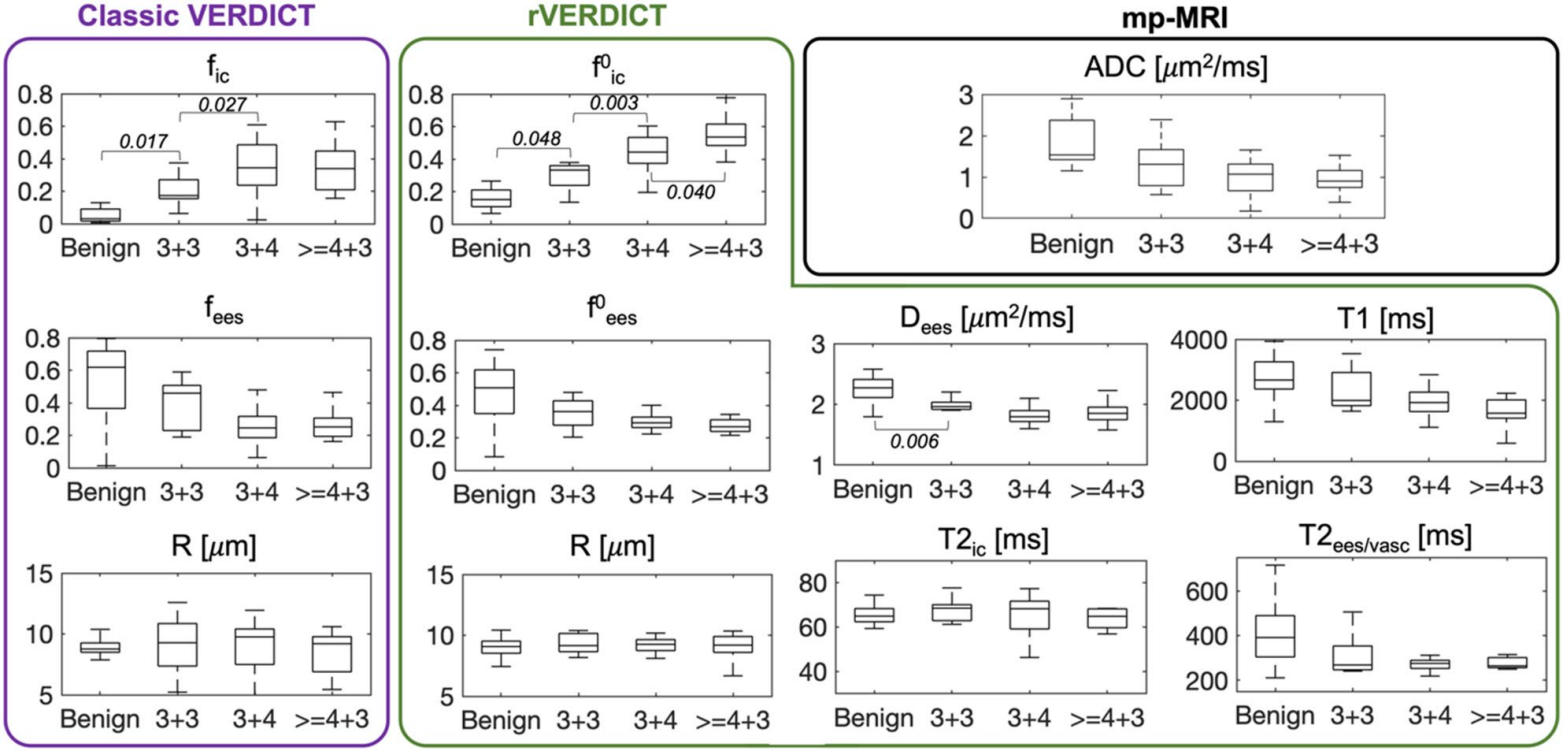
Box-and-whisker plots of the classic VERDICT, rVERDICT parameters and ADC from mp-MRI as a function of the Gleason grade groups. Only the differences with Bonferroni corrected *p* < 0.05 are considered significant and the corresponding *p* values reported.

Figure 6 reports the box-and-whisker plots of the rVERDICT and VERDICT parameters as well as ADC from mp-MRI for four Gleason grades groups (benign, 3 + 3, 3 + 4, ≥ 4 + 3). We replicated the previously reported ability of VERDICT f_ic_ to distinguish Gleason score 3 + 3 from 3 + 4 (*p* = 0.027), but not Gleason 3 + 4 from ≥ 4 + 3 (*p* > 0.05), in agreement with^16^. We also replicated the results concerning the ADC from mp-MRI: the ADC does not discriminate between 3 + 3 and 3 + 4 (*p* > 0.05), nor 3 + 4 and ≥ 4 + 3 (*p* > 0.05). rVERDICT ${\text{f}}^{0}_{{{\text{ic}}}}$ improved the discrimination of Gleason score 3 + 3 from 3 + 4 (*p* = 0.003) and additionally showed discrimination of Gleason 3 + 4 from ≥ 4 + 3 (*p* = 0.040). Noteworthy, VERDICT f_ic_ can distinguish the PCa lesion from benign tissue better than rVERDICT ${\text{f}}^{0}_{{{\text{ic}}}}$ (*p* = 0.017 vs. *p* = 0.048), although both parameters enable statistically significant discrimination.

### rVERDICT parametric maps for different Gleason grades

**Figure 7:**
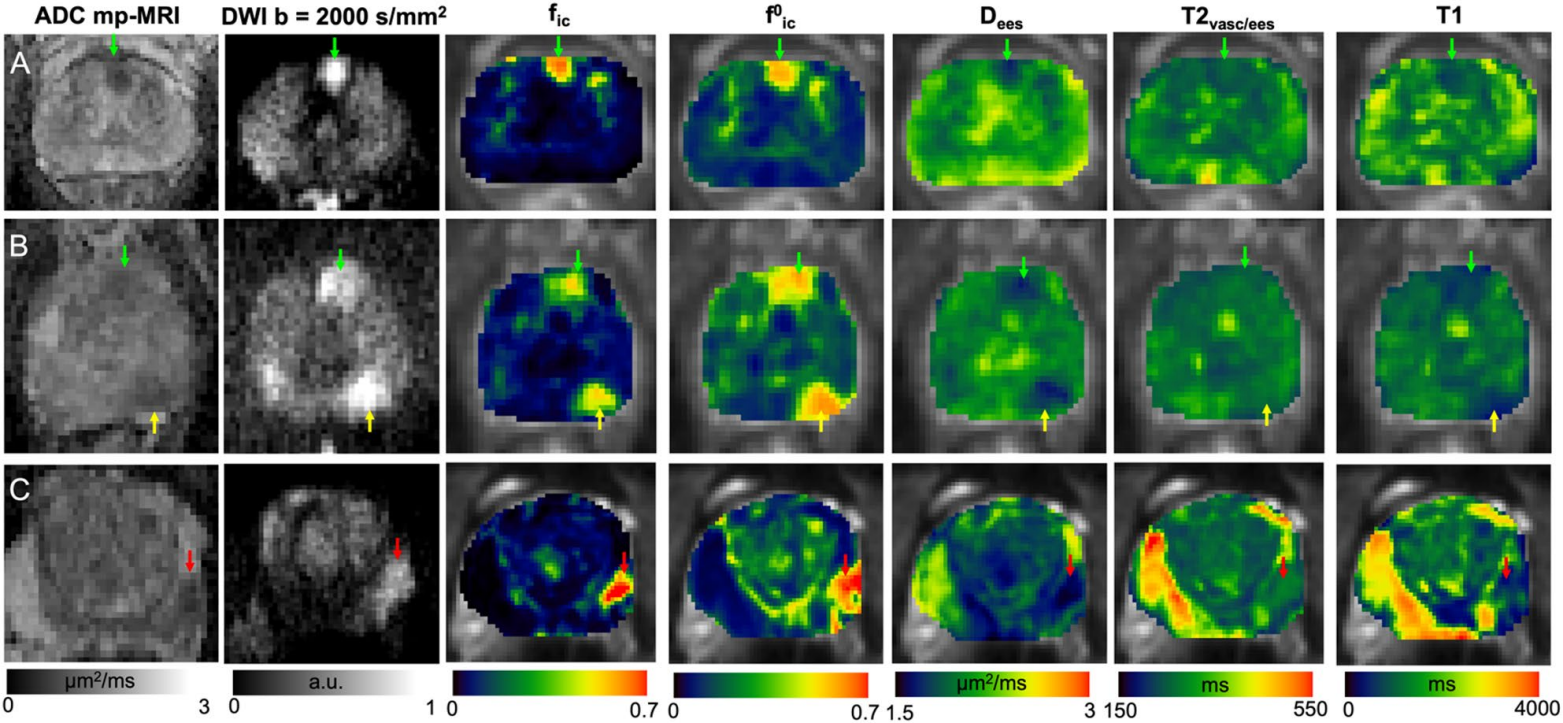
(**A**–**C**) Apparent diffusion coefficient (ADC) maps from multi-parametric MRI (mp-MRI); diffusion-weighted image (DWI) at b = 2000s/mm^2^, classic VERDICT intracellular volume fraction f_ic_ and rVERDICT maps (intracellular volume fraction ${\text{f}}^{0}_{{{\text{ic}}}}$; extracellular-extravascular apparent diffusion coefficient D_ees_; vascular/extracellular-extravascular T2 relaxation time T2_vasc/ees_; T1 relaxation time) for three exemplar patients with different PCa: (**A**) age late 50’s, PSA 4.78 Gleason 3 + 3 MCCL 7 mm; lesion in the anterior gland; (**B**): age late 60’s, PSA 5.21 left posterior lesion Gleason 3 + 4 MCCL 14 mm, left anterior lesion Gleason 3 + 3; (**C**): age late 60’s, PSA 8.68 Gleason 4 + 3, MCCL 10 mm; lesion in the left peripheral zone. Green arrows indicate Gleason grade 3 + 3, yellow arrows Gleason grade 3 + 4 and red arrows Gleason grade 4 + 3.

Parametric maps for three exemplar cases are shown in Fig. 7 to demonstrate lesions with Gleason score 3 + 3 (Fig. 7A, green arrow), 3 + 4 (Fig. 7B, yellow arrow) and 4 + 3 (Fig. 7C, red arrow) on the DWI at b = 2000s/mm^2^, ADC from mp-MRI, VERDICT f_ic_ and rVERDICT ${\text{f}}^{0}_{{{\text{ic}}}}$, apparent extracellular-extravascular diffusivity D_ees_, T2_vasc/ees_ and apparent T1 relaxation time. A direct comparison of all the VERDICT parametric maps with the corresponding ones from rVERDICT for the representative participant in Fig. 7B is in Supplementary Fig. S1.

## Discussion

In this work, we propose a new prostate model called relaxation-VERDICT (rVERDICT) that provides joint estimation of relaxation and diffusion parameters, such as the intracellular T2 relaxation time (T2_ic_) and the intracellular signal fraction (f_ic_). Our hypothesis is that a unifying model capturing both relaxation and diffusion effects would enhance the accuracy of model parameters estimation and consequently the Gleason grade discrimination. As prostate histological components differ between Gleason grades^45^, we expect diffusion parameters, and in particular the f_ic_ from classic VERDICT that correlates with epithelial volume fraction^15,18^, to provide high biologic specificity to Gleason grade. However, classic VERDICT can only achieve discrimination of Gleason 3 + 3 from 3 + 4^16^. Gleason discrimination for higher scores like 3 + 4 from ≥ 4 + 3 is also important, as 4 + 3 cancers are associated with a three-fold increase in lethal PCa compared to 3 + 4 cancers^6^. Here we hypothesise that rVERDICT can compensate for any relaxation-induced bias that may be reducing the accuracy of classic VERDICT estimates, enabling robust identification and discrimination of Gleason 4 components.

Our results (Fig. 6) support our hypotheses, showing that the new information obtained from rVERDICT enables the discrimination of Gleason 3 + 3, 3 + 4 and ≥ 4 + 3. Most importantly, on the previously unattainable differentiation of Gleason grade 3 + 4 and ≥ 4 + 3, rVERDICT achieved statistical significance (*p* = 0.040 for ${\text{f}}^{0}_{{{\text{ic}}}}$). The improved performance of rVERDICT over VERDICT is probably due to the compensated relaxation-induced biases on the signal fractions and the extra information on the relaxation times. Additional numerical simulations we performed showed that rVERDICT reduces the error of classic VERDICT on (f_ic_, f_ees_, f_vasc_) by respectively (65, 93, 12) percentage points for the ex vivo and (64, 83, 20) percentage points for the in vivo case (see Supplementary Material and Supplementary Fig. S4).

The clinical utility of rVERDICT is demonstrated by the repeatability and fitting performance results (Figs. 3 and 4), alongside the application of rVERDICT to data acquired with a clinical scanner part of a clinical trial (registered with ClinicalTrials.gov identifier NCT02689271). The ${\text{f}}^{0}_{{{\text{ic}}}}$ from rVERDICT achieved higher repeatability (R^2^ = 0.98; CV = 7%) compared to VERDICT f_ic_ (R^2^ = 0.83; CV = 27%, from^44^), suggesting that we can achieve greater repeatability by removing confounds through disentangling relaxation from diffusion parameters. Additionally, the fitting approach based on DNN provides accurate and precise estimates for all the rVERDICT parameters with dramatic reduction of the processing time (~ 2 min vs. ~ 2 h using non-linear least squares minimization, with similar stability), enabling on-the-fly rVERDICT map generation. This is a critical point that further enables clinical translation of the technique, as precision, robustness and computational cost are among the main issues that forbid advanced microstructural imaging from clinical adaptation. Moreover, the DNN proposed in this study is highly generalizable as it can be readily (and quickly) trained using simulated synthetic signals from any arbitrary DW-MRI acquisition protocol.

An advantage of rVERDICT is the possibility to obtain simultaneously diffusion and relaxation properties of prostate tissue using only a 12-min DW-MRI acquisition that can be readily implemented on any clinical scanner. We showed that estimates of T2 and T1 relaxation times with rVERDICT match those from independent measurements and literature. The T1 relaxation estimates had lower values within tumour lesions than in benign tissue (mean 1576 vs. 2754 ms, respectively, *p* = 0.003), in line with estimates obtained using independent T1 measurements^26–28,41^. The T2 relaxation estimates T2_ic_ within tumours were similar for benign tissue (mean 61 vs. 67 ms, respectively, *p* > 0.05) and T2_vasc/ees_ within tumour lesions were lower than in benign tissue (mean 300 vs. 383 ms, respectively, *p* = 0.023), in agreement with literature^26,27,30,32,33,42,43^. For seven patients in our cohort, we performed an independent multi-TE acquisition which allowed for a direct comparison of the estimated T2 values using the two methods. Results showed that the rVERDICT T2 values were not statistically different (*p* > 0.05) from the independent T2 measurements (Fig. 5). Noteworthy, measurements of T2 relaxation times using multi-echo spin-echo acquisitions may be affected by stimulated and indirect echoes^46^. However, our estimated relaxation times for a two-pool model are in agreement with the literature, including studies that use single-echo spin-echo acquisitions^42^ and MR fingerprinting^41^. Therefore, we believe the bias is negligible in our case. Finally, although multi-TE acquisitions have the advantage of offering MR images with higher resolution and fewer artefacts compared to DW-MRI (which could partly explain the differences between the corresponding distributions in Fig. 5), diffusion-based techniques give us unique insight into microstructure.

To demonstrate the potential of rVERDICT for improving PCa diagnosis, we present three example cases in Fig. 7. The new information provided by rVERDICT maps can help improve the ability of identifying and distinguishing Gleason grades of PCa lesions in cases where both the ADC map and the high b-value DWI already show clear contrast (Fig. 7A) and most importantly when these conventional measures provide ambiguous information (Fig. 7B and C). The direct comparison of rVERDICT parametric maps with the classic VERDICT counterparts (also in Supplementary Fig. S1) showed generally higher f_ic_ and lower f_ees_ estimates, especially in the cancerous areas, in agreement with the simulations in Supplementary Fig. S4 and the results in Fig. 6. Although the R maps show differences between the two methods (Supplementary Fig. S1), the estimated cell radius from both rVERDICT and VERDICT ranges between ~ 8 and ~ 11 μm, in agreement with^15,44,47^. A correlation analysis of pairs of rVERDICT parameters at the voxel and patient levels highlighted moderate to strong correlations between some of the rVERDICT model parameters (see Supplementary Materials and Supplementary Fig. S6), which agree with the existing literature and can be explained by the known histopathology of PCa. We found a strong negative correlation between ${\text{f}}_{{{\text{ic}}}}^{0}$ and f_ees_^0^ that can be explained by the fact that in real tissue more intracellular space (higher ${\text{f}}_{{{\text{ic}}}}^{0}$) leads necessarily to less extracellular space (lower ${\text{f}}_{{{\text{ees}}}}^{0}$); and vice versa. We found moderate to strong negative correlation between ${\text{f}}_{{{\text{ic}}}}^{0}$ and T1 and ${\text{f}}_{{{\text{ic}}}}^{0}$ and T2 (both T2_vasc/ees_ and T2_ic_) which agrees with our hypothesis that ${\text{f}}_{{{\text{ic}}}}^{0}$ is mostly capturing the epithelium contribution in PCa. Indeed, several studies have shown that T1 and T2 relaxation values correlate negatively only with density of epithelium in PCa^32,36,48–51^. The negative correlation between ${\text{f}}_{{{\text{ic}}}}^{0}$ and T1 can be explained, as suggested in^51^, since cancer disrupts the glandular spaces containing fluid most likely having a long T1. Consequently, in cancer—especially for higher Gleason grades—the tissue containing the disrupted ducts might contain many more cells and less long-T1 fluid, and therefore have an average T1 which is shorter, while an average ${\text{f}}_{{{\text{ic}}}}^{0}$ which is higher. Likewise, this could also explain the observed positive correlation between ${\text{f}}_{{{\text{ees}}}}^{0}$ and T1; ${\text{f}}_{{{\text{ees}}}}^{0}$ and D_ees_; and the negative correlation between ${\text{f}}_{{{\text{ic}}}}^{0}$ and D_ees_.

In comparison to recent diffusion-relaxation techniques proposed for prostate tissue characterization^30,32,33^, rVERDICT has several differences. Firstly, rVERDICT (as VERDICT) explicitly models and quantifies the contribution of vasculature, which is instead neglected in^30,32,33^. While the current literature is still controversial about vascularization and cancer aggressiveness for prostate^52–55^, there is general agreement that increased angiogenesis is an important factor in determining tumour development and prognosis^55^. For cases with aggressive cancer, showing significant vascularisation or neovascularisation, the ${\text{f}}^{0}_{{{\text{vasc}}}}$ map could potentially provide higher discriminative power and/or aid early diagnosis. Here, we estimated ${\text{f}}_{{{\text{vasc}}}}^{0}$ values ~ 10–25% in the benign tissue and ~ 5–15% in PCa, in agreement with histological examinations^11^ and alternative MRI estimations (e.g., intravoxel incoherent motion imaging—IVIM^10,56^). Also, unlike^32,33^ and similarly to^47^, we explicitly model restriction by considering a compartment of water restricted in the intracellular space, accounting for epithelium. We note that in our model, the signal contribution from stroma is likely captured by the extravascular/extracellular compartment (Supplementary Fig. S3). Previous investigations and histological validations have demonstrated the validity of these assumptions^15,18^, showing good agreement between the VERDICT estimated f_ic_ and f_ees_ with histological measurements of epithelium and stroma plus lumen volume fraction, respectively. We note that the results reported in^33^ also suggest that the stroma component does not change significantly in prostate cancer. In rVERDICT (as in VERDICT and^47^) we also model the effective apparent size R of the cellular component that is not modelled in^32,33^ and is only indirectly estimated in^30^. Our simulations showed no strong correlation between any pairs of the estimated rVERDICT model parameters, confirming that rVERDICT is not over-parametrized and reports on independent properties of the tissue (see Supplementary Materials and Supplementary Fig. S6).

There are several opportunities for further improvement in the future. The analysis presented here was performed on retrospective data with an acquisition protocol optimised for classic VERDICT probing a limited range of TE and TR values. This mostly compromises the sensitivity to long T2 values (T2_vasc/ees_) and the reproducibility of measured T1 values. However, we showed that our T2 estimates are still in agreement with independent T2 measurements that cover a wider acquisition parameter space. Also, our simulation results in Supplementary Fig. S5 suggest an error in the estimated values of the long T2 within ± 5% of the true value. Future work will explore optimization of the VERDICT MRI acquisition to explicitly account for T1 and long T2 relaxation times, and direct comparison with independent measurements of T1. Additionally, this study analysed only 44 patients for whom the biopsy results were available. This resulted in limited/unbalanced Gleason grades groups, hampering the possibility to examine differences in diagnostic performance (e.g. with comparison of areas under the receiver operating characteristic curve). However, we were still able to draw significant differences and demonstrate the potential of rVERDICT. Further study will include rVERDICT analysis on larger cohorts. From a modelling perspective, rVERDICT (as VERDICT) does not account for the effect of exchange and diffusion time dependence of water diffusivity^30,31^. However, the diffusion time used in this study was between 22 and 36 ms, a range for which previous studies have shown negligible effects due to permeability and time dependence^30,31,57^. The contribution of stroma is not explicitly modelled by rVERDICT and, as in VERDICT, it is assumed to contribute to the extracellular-extravascular compartment. Given recent experimental evidence that T2 values of stroma are closer to epithelium than lumen^32,33^, we assessed using numerical simulations how assuming a unique average T2 for stroma and lumen could affect the accuracy of estimating ${\text{f}}^{0}_{{{\text{ic}}}}$. We found that this assumption leads on average to underestimate the true epithelial signal fraction by ≤ 20 percentage points (both ex vivo and in vivo). This bias reduces to ≤ 10 percentage points when high SNR (≥ 100) can be achieved (Supplementary Figs. S3 and S4). Future work can explore the possibility of including these effects in the model and potentially estimating other tissue properties such as cell membrane permeability and isolate and estimate the stroma contribution. Finally, the DNN used for model parameters estimation does not account for any spatial relationship between voxels. Future work may consider using a CNN based architecture, which would naturally account for such spatial relationship and regularize the fitting of rVERDICT to the data, potentially providing some additional benefits in terms of accuracy and robustness to noise.

## Conclusions

In conclusion, rVERDICT with machine learning allows for accurate, fast and repeatable microstructural estimation of both diffusion and relaxation properties of prostate cancer. This enables differentiation of Gleason grades, potentially allowing the utilisation of rVERDICT for clinical use and improved diagnosis.

## Methods

### Patient population and study design

This study was performed with local ethics committee approval embedded within the INNOVATE clinical trial^19^. Ethical approval for the prospective INNOVATE study (ClinicalTrials.gov: NCT02689271) was granted by the UK Research Ethics Committee (ref: 15/LO/0692). The trial is registered with ClinicalTrials.gov identifier NCT02689271. The study abides by the principles of the Declaration of Helsinki and the UK Research Governance Framework version 2. INNOVATE received UK Research Ethics Committee approval on 23rd December 2015 by the NRES Committee London—Surrey Borders with REC reference 15/LO/0692.

A participation flow diagram is shown in Fig. 1. 72 men (median age = 64.8 years; range = 49.5–79.6 years) were recruited and provided informed written consent. The inclusion criteria were: (1) suspected PCa or (2) undergoing active surveillance for known PCa. Exclusion criteria included: (1) previous hormonal, radiation therapy or surgical treatment for PCa and (2) biopsy within 6 months prior to the scan. All patients underwent mp-MRI in line with international guidelines^58^ on a 3 T scanner (Achieva, Philips Healthcare, Best, Netherlands) supplemented by VERDICT DW-MRI (the clinical DCE part of mp-MRI was performed last after the VERDICT MRI).

After the clinical mp-MRI and VERDICT DW-MRI, 44 participants underwent targeted transperineal template biopsy of their index lesion as clinically indicated. The index lesion was defined as the highest scoring lesion identified on mpMRI with Likert scores (3–5). The mp-MRI was used to guide cognitive targeted template biopsy (performed by experienced urologists). Specialist genitourinary pathologists (A.F. and M.R) evaluated histological specimens stained with haematoxylin and eosin from the biopsy cores and assigned each biopsy core a Gleason grade (Fig. 1).

### DW-MRI acquisition

The VERDICT protocol, adapted from^44^, acquires DW-MRI data using pulsed-gradient spin echo (PGSE) at five combinations (b; δ; Δ; TE; TR) of b‐values b (in s/mm^2^), gradient duration δ, separation Δ, echo time TE and repetition time TR (in ms): respectively, (90; 3.9; 23.8; 50; 2482); (500; 11.4; 31.3; 65; 2482); (1500; 23.9; 43.8; 90; 2482); (2000; 14.4; 34.4; 71; 3945); (3000; 18.9; 38.8; 80; 3349), in three orthogonal directions using a cardiac coil. For each combination, a separate b = 0 image was acquired, providing a total of ten different measurements. For b < 100 s/mm^2^ the number of averages (NAV) = 4 and for b > 100 s/mm^2^ NAV = 6; voxel size = 1.3 × 1.3 × 5 mm; matrix size = 176 × 176; average signal-to-noise ratio (SNR) = 35; scan duration = 12′25″.

### Scan-rescan acquisition

Scan-rescan repeatability of the VERDICT DW-MRI acquisition protocol was performed in five participants (median age = 68 years; range = 50–79 years) randomly chosen among the first 40 participants recruited for the INNOVATE study^19^, thus sharing the same inclusion/exclusion criteria. Participants were imaged twice, taking them out of the scan with less than 5-min break in between the scans.

### T2-relaxometry MRI

A multi-TE acquisition was acquired for an independent estimate of the multiple T2 relaxation times for seven participants (median age = 65 years; range = 49–79 years), randomly chosen among those recruited for the INNOVATE study^19^, thus sharing the same inclusion/exclusion criteria. The details of the acquisition are in Supplementary Materials.

### DW-MRI pre-processing

The pre-processing included denoising using MP-PCA^59^ as implemented within MrTrix3^60^ ‘dwidenoise’; correction for Gibbs ringing^61^ with custom code in MATLAB (The Mathworks Inc., Natick, Massachusetts, USA); correction of motion artefacts and eddy current distortions by mutual-information rigid and affine registration using custom code in MATLAB.

### T2-relaxometry MRI pre-processing

The pre-processing included only registration of the images at each TE to the image at the first TE, using the same mutual-information rigid registration used for the DW-MRI pre-processing.

### VERDICT model

The VERDICT model^11^ is the sum of three parametric models, each describing the DW-MRI signal in a separate population of water from one of the three compartments: S_ic_ comes from intracellular water (including epithelium), modelled as restricted diffusion in spheres of radius R and intra-sphere diffusivity D_ic_ = 2 μm^2^/ms (value that minimised fitting error averaged over all PZ voxels and in agreement with recent ultra-short diffusion-time measurements^47^); S_ees_ comes from extracellular-extravascular water adjacent to, but outside cells and blood vessels (including stroma and lumen), modelled as Gaussian isotropic diffusion with effective diffusivity D_ees_ = 2 μm^2^/ms (value that minimised fitting error averaged over all PZ voxels and in agreement with alternative measurements^34,47^); and S_vasc_ arises from water in blood undergoing microcirculation in the capillary network, modelled as randomly oriented sticks with intra-stick diffusivity D_vasc_ = 8 μm^2^/ms, which also accounts for any intra-voxel incoherent motion effects. The total MRI signal for the VERDICT model is:

1 $$S\left( b \right)/S_{0} = f_{vasc} S_{vasc} \left( {D_{vasc} , b} \right) + f_{ic} S_{ic} \left( {D_{ic} ,R,b} \right) + f_{ees} S_{ees} \left( {D_{ees} ,b} \right)$$

where f_i_ is the proportion of signal from water molecules in population i = vasc;ic;ees, ${f}_{vasc}+{f}_{ic}+{f}_{ees}=1$ and ${S}_{0}$ is the b = 0 signal intensity. We refer to the original VERDICT works^11,14^ for the specific expressions for S_vasc;ic;ees_, and the choice and interpretation of the model parameters.

### rVERDICT model

Mathematically, the rVERDICT model is

2 $$S\left( {b, TE, TR} \right) = S_{0} \left( {1 - e^{{ - \frac{TR}{{T1}}}} } \right)\left[ {f_{vasc}^{0} e^{{ - \frac{TE}{{T2_{vasc/ees} }}}} S_{vasc} \left( {D_{vasc} , b} \right) + f_{ic}^{0} e^{{ - \frac{TE}{{T2_{ic} }}}} S_{ic} \left( {D_{ic} ,R,b} \right)} \right.\left. { + f_{ees}^{0} e^{{ - \frac{TE}{{T2_{vasc/ees} }}}} S_{ees} \left( {D_{ees} ,b} \right)} \right]$$

where we adopt the same terminology as VERDICT, but here the signal fractions ${\text{f}}^{0}_{{\text{i}}}$, where i = vasc;ic;ees, avoid the bias in the corresponding VERDICT parameters from MR relaxation tissue properties^25^. Like in VERDICT, we fix D_ic_ = 2 μm^2^/ms and D_vasc_ = 8 μm^2^/ms, but we leave D_ees_ as a free parameter to be estimated from the data to impose less constraints than the original VERDICT and because there have been studies using the classic VERDICT that also estimated D_ees_ with promising biomarker potential^44^. The other free parameters to be estimated from the data are: the T2 relaxation of the intracellular compartment T2_ic_; the T2 relaxation of vascular and extracellular/extravascular compartments T2_vasc/ees_; the T1 relaxation contribution from the whole tissue; the S_0_ signal at (b = 0, TE = 0, TR = inf); the signal fractions ${\text{f}}^{0}_{{{\text{i}} = {\text{ic}},{\text{ees}}}}$ and the apparent MR cell radius R.

### VERDICT and rVERDICT analysis of DW-MRI data

We obtained quantitative maps from both VERDICT and rVERDICT by fitting respectively Eqs. (1) and (2) to the VERDICT DW-MRI data, using the signal averaged across the three gradient directions.

The VERDICT model has three free model parameters (f_ees_, f_ic_, R) that we estimate by fitting Eq. (1) to the five DW-MRI measurements at nonzero b values, normalized by their corresponding b = 0 measurements. The f_vasc_ = 1 − f_ic_ − f_ees_; the cellularity = f_ic_/R^3^.

The rVERDICT model has eight free parameters (S_0_, T1, T2_ic_, T2_vasc/ees_, ${\text{f}}^{0}_{{{\text{ees}}}}$, ${\text{f}}^{0}_{{{\text{ic}}}}$, R, D_ees_) that we estimate by fitting Eq. (2) to the ten DW-MRI measurements: the five nonzero b value measurements and their corresponding five b = 0 measurements. Hence, unlike VERDICT, for rVERDICT we exploit the TE and TR dependence of the five b = 0 measurements to estimate the T2 and T1 relaxation times.

For fast inference, we performed the fitting using a DNN comprised of three fully connected layers^62–64^. We trained the DNN in a supervised fashion using fully synthetic signals generated using Eqs. (1) or (2) with the addition of Rician noise, according to the same imaging protocol used to acquire the real VERDICT MRI data (further details in Supplementary Materials). The creation of the training set and training of the DNN (to be done only once) took ~ 100 s using 4 threads on Intel Core i7 processor at 2.4 GHz; the prediction of the trained DNN for the whole unmasked DW-MRI dataset (~ 5 × 10^5^ voxels) took ~ 35 s, for each subject. For comparison, the fitting through the non-linear least squared minimization implemented in the MATLAB’s ‘nonlincon’ function took ~ 8000 s (with initial guess chosen through grid-searching).

### DNN model fitting assessment

To assess the accuracy and precision of the DNN estimator for a complex model as rVERDICT we performed numerical simulations with known ground-truth. First, to guarantee generalizability, we simulated signals from model parameters combinations covering the whole parametric space, and not just the subset of realistic prostate tissue combinations (further details in Supplementary Materials). The DNN was used to predict the rVERDICT model parameters from these signals and we evaluated accuracy and precision in terms of bias and dispersion of the prediction compared to the known ground-truth. For a realistic combination of the model parameters, mirroring values reported for PCa in PZ and TZ^32,33,44^ (T1 = 2700 ms; T2_ic_ = 70 ms; T2_vasc/ees_ = 530 ms; ${\text{f}}^{0}_{{{\text{ees}}}}$ = 0.40; ${\text{f}}^{0}_{{{\text{ic}}}}$ = 0.40; R = 8 μm; D_ees_ = 2 μm^2^/ms), we evaluated the stability of the fit with respect to possible degeneracy and local minima by comparing the distribution of predicted values for each model parameter with the known ground-truth, when the other six parameters were varied taking four values linearly spaced within their biophysically plausible ranges (i.e. 4^6^ = 4096 different noisy realizations). As benchmark, we compared the performance of our DNN model with the MATLAB’s ‘nonlincon’ function, using a grid-search algorithm for the initial guess.

### T2 estimates assessment

To assess the differences between the T2 relaxation times estimated using rVERDICT and those using independent multi-TE acquisition (details in Supplementary Materials), we compared the distribution of T2 values estimated by the two methods for all the voxels within the prostate volume, and corresponding median, 25th and 75th percentiles. Statistically significant differences were assessed by a two-sided Wilcoxon rank sum test.

Given the short maximal TE used in our sequence (90 ms), we also performed numerical simulations to assess the accuracy of the estimation of long T2 values (details in Supplementary Materials).

### Regions-of-interest definition

Two board‐certified experienced radiologists (reporting more than 2000 prostate MR scans per year) manually placed ROIs on the VERDICT DW-MRI, guided by the standard mp-MRI index lesions and confirmed the ROIs with the biopsy results (further details in Supplementary Materials). The VERDICT MRI dataset was not explicitly co-registered to the mp-MRI dataset to avoid bias from errors in the registration.

### Gleason grade differentiation

To assess the ability of rVERDICT to discriminate between Gleason grade groups and compare with VERDICT and ADC from mp-MRI, analysis of variance with Bonferroni multiple comparisons correction was performed to determine statistically significant differences between four groups: benign, Gleason grades 3 + 3, 3 + 4, ≥ 4 + 3 (for consistency with previous studies^16^), for all rVERDICT and VERDICT parameters and ADC from mp-MRI (considering all the ROIs).

### Scan-rescan repeatability

We quantified repeatability using the adjusted coefficient of determination R^2^ between each estimated model parameter in the first scan with the estimates from the second scan, considering all the voxels within each of the n = 179 ROIs. We used subject-specific ROIs instead of whole prostate statistics to remove potential bias due to deformation and different position of the prostate between the two scans. We used Bland–Altman plots and computed the coefficient of variation (CV) as standard deviation over the mean and the intraclass correlation coefficient (ICC), calculated for two-way mixed effects, single measurement, with absolute agreement.

## Supplementary Information

Supplementary Information.

## Data Availability

The code to perform VERDICT and rVERDICT analysis will be publicly available at https://github.com/palombom upon acceptance of the paper. The data used in this study are available from the corresponding author on reasonable request.
