# Supplementary material for: Joint estimation of relaxation and diffusion tissue parameters for prostate cancer with relaxation-VERDICT MRI
Source: Sci Rep. 2023 Feb 21;13:2999. doi: 10.1038/s41598-023-30182-1 (PMC9943845; doi:10.1038/s41598-023-30182-1)
Supplement: Supplementary file 1 — Supplementary Information. [file 41598_2023_30182_MOESM1_ESM.docx]

**Supporting Materials**


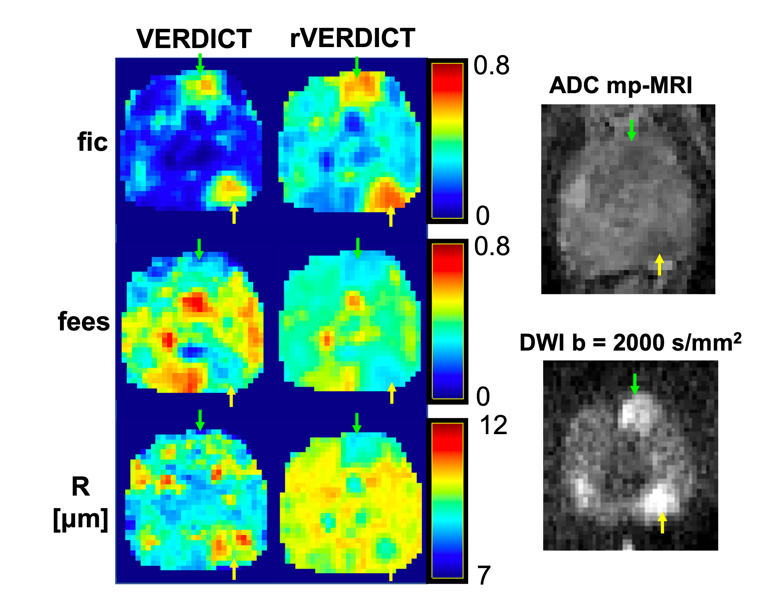


***Figure S1.*** *Exemplar comparison of f_ic_, f_ees_, and R maps from classic VERDICT and corresponding ones from rVERDICT for the patient in Figure 7b from the main text: age late 60’s, PSA 5.21 left posterior lesion Gleason 3+4 MCCL 14 mm (yellow arrow), left anterior lesion Gleason 3+3 (green arrow). We observe generally higher f_ic_ estimates, and lower f_ees_ estimates, especially in the cancerous areas, in good agreement with the simulations results reported in Figure S3 and Figure 6 from the main text. Regarding R, the estimates of cell radius from both rVERDICT and VERDICT ranges from ~8 to ~11 μm, but their spatial distribution over the whole prostate tissue is different between the two methods.*

**Conventional ADC measurements from mp-MRI**

All participants were scanned with a diffusion-weighted echo-planar imaging sequence to estimate the apparent diffusion coefficient (ADC) maps (as part of mp-MRI) with: TR/TE=2753/80 ms; slice thickness 5 mm; no interslice gap; acquisition matrix 168 × 169 mm; b=0, 150, 500, 1000 s/mm2; and six directions per b value. The total imaging time was 5’16”. ADC maps were calculated by using all b values except b=0 to reduce perfusion effects^1^ and were calculated with the Camino Diffusion MRI toolkit^2^.

**Multi-TE T2-relaxometry MRI acquisition**

The multi-TE acquisition for independent estimation of T2 relaxation times in prostate consisted of a multi-echo spin‐echo sequence with an echo spacing of 31.25 msec and TR=8956 msec. The other imaging parameters were: number of echo times=32; field of view (FOV)=180×180 mm; acquired voxel size=2×2×4 mm; scan duration=5’50”.

**Details on Model fitting with deep neural network (DNN)**

In this work, we performed the model fitting using the ‘MLPregressor’ implemented in Python scikit-learn 0.23 ([https://scikit-learn.org](https://scikit-learn.org/stable/)). The input of our DNN is the signal in each MRI voxel, i.e. a vector whose elements are the DW-MRI signals for each of the ten measurements at the different b, TE and TR combinations. Therefore, each MRI voxel is considered as an independent vector of measurements, and no spatial relationship between voxels is considered, neither during training nor during prediction. The DNN then outputs a vector of eight rVERDICT model parameters (or 3 model parameters in the case of classic VERDICT implementation). The DNN consists of three fully-connected hidden layers with 150 units, each characterised by a linear matrix operation followed by element-wise rectified linear unit function (ReLU), and a final regression layer with the number of output units equal to the number of tissue parameters to be estimated. The DNN is optimised by backpropagating the mean squared error (MSE) between ground truth model parameters and DNN predictions. We performed the optimisation with the adaptive moment estimation (ADAM) method^3^ for 1000 epochs (adaptive learning rate with initial value of 0.001; one update per mini-batch of 100 voxels; early stopping to mitigate overfitting; and momentum = 0.9) on 100,000 synthetic DW-MRI signals (split into 80% for training and 20% for validation). We generate the synthetic DW-MRI signals using equation [2] (or similarly [1]) with different values for the model parameters randomly chosen between biophysical plausible intervals: S_0_ = [0, maximum b=0 intensity x 2], T1 = [10, 4000] ms, T2_ic_ = [1, 150] ms, T2_vasc/ees_ = [150, 800] ms, f_ees_ and f^0^_ees_ = [0.01, 0.99], f_ic_ and f^0^_ic_ = [0.01, 0.99], R = [0.01, 15] μm and D_ees_ = [0.5, 3] μm^2^/ms. Note that we chose a value of 150 ms to separate the short and long T2 components during training, according to the rVERDICT’s assumptions. We also added Rician noise corresponding to SNR = 35 to consider experimental noise effect. For the final parameter computation, we used the DNN at the epoch with minimum validation loss. Exemplar training data are shown in the **Supplementary Figure S2**.


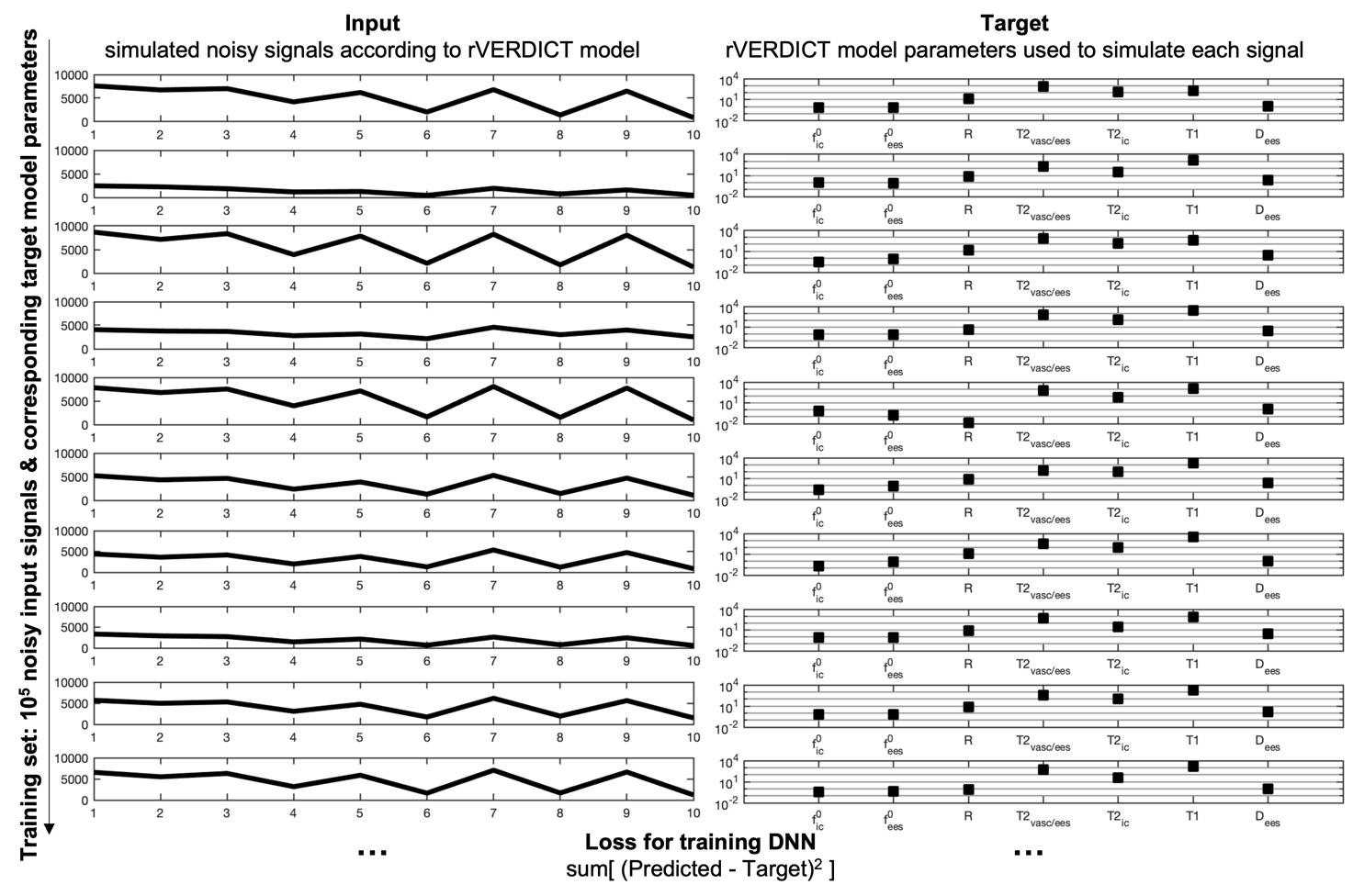


***Figure S2.*** *Exemplar training data and corresponding ground truth targets for the eight rVERDICT model parameters.*

**DNN model fitting assessment**

To assess the accuracy and precision of the DNN estimator, we generated synthetic DW-MRI signals with equation [2] in the main text and all the possible combinations of the seven model parameters using four values linearly distributed in the intervals: T1 = [10, 4000] ms, T2_ic_ = [1, 150] ms, T2_vasc/ees_ = [150, 800] ms, f^0^_ees_ = [0.01, 0.99], f^0^_ic_ = [0.01, 0.99], R = [0.01, 15] μm and D_ees_ = [0.5, 3] μm^2^/ms. These define a 7-dimensional grid comprised of 4^7^ = 16,384 unique combinations of the seven model parameters and uniformly covering the whole parametric space of rVERDICT model. We also added Rician noise corresponding to SNR = 35 to match experimental noise conditions.

**Multi-TE T2-relaxometry MRI analysis**

To provide an independent T2 estimation of the relaxation times T2_ic_ and T2_vasc/ees_ assumed in rVERDICT, we fitted the following equation to the multi-TE data:

$M\left( TE \right)=A_{ic}\exp\left( -\frac{TE}{{T2}_{ic}} \right)+A_{vasc/ees}\exp\left( -\frac{TE}{{T2}_{vasc/ees}} \right)+\varepsilon$ [S1]

where A_i_ are the relative signal intensities of the compartment i=ic;vasc/ees, T2_i_ are the corresponding T2 values, with T2_ic_<T2_vasc/ees_, and ε is a plateau constant, accounting for non-zero noise floor in magnitude images and found to improve significantly the T2 estimates^4^.

For the fitting of equation [S1] to the T2-relaxometry MRI data, we used the same DNN described in the previous section, trained on 100,000 synthetic signals obtained using equation [S1] with different random values between reasonable intervals: A_i=ic,vasc/ees_ = [0, maximum b=0 intensity x 2], T2_ic_ = [1, 150] ms, T2_vasc/ees_ = [150, 800] ms, and ε = [0.01, 0.20]. We added Rician noise with SNR = 25 to match experimental noise conditions.

**Assessing rVERDICT assumptions for the extracellular-extravascular compartment**

In this work we proposed rVERDICT as extension of classic VERDICT to account for compartmental relaxation properties. As such, in rVERDICT we keep the same assumptions as in classic VERDICT. One of these is that the stroma is associated to the extracellular-extravascular compartment. In the original VERDICT work^5^, this choice was motivated by experimental evidence that the diffusion-weighted MR signal from diffusion restricted in spheres highly correlates with epithelial tissue compartment and it was validated with histology^6,7^. Since VERDICT is a three-compartment model having one compartment explicitly modelling vasculature, the only other compartment able to account for tissue contributions different from diffusion restricted in spheres (e.g. stroma) is the extracellular-extravascular compartment. Hence, stroma and lumen were assumed to both contribute to the signal of this compartment. Here we performed numerical simulations showing that indeed the overall diffusion-weighted signal decay from stroma is closer to that of lumen, further support this assumption.

We used a complete four-compartment model:

$$S_{tot}= S_{epithelium}+S_{stroma}+S_{lumen}+S_{vasculature}=$$

$= f_{epithelium}e^{-\frac{TE}{{T2}_{epithelium}}}e^{-b{ADC}_{epithelium}}+f_{stroma}e^{-\frac{TE}{{T2}_{stroma}}}e^{-b{ADC}_{stroma}}+f_{lumen}e^{-\frac{TE}{{T2}_{lumen}}}e^{-b{ADC}_{lumen}}+f_{vasculature}e^{-\frac{TE}{{T2}_{vasculature}}}e^{-b{ADC}_{vasculature}}$ [S2]

with the TE and b values used in our experiments and the tissue model parameters reported by previous studies at 3T for PCa in the PZ (midgland) in both ex vivo (extrapolated from^8^) and in vivo (extrapolated from^9^). For the vasculature component, we used reference values from^10^. These values are summarized in the table below and the results of the simulated signals are reported in the Figure S2, together with the mean squared error computed between S_epithelium_ and S_stroma_, mse_epithelium-stroma_, and S_lumen_ and S_stroma_, mse_lumen-stroma_.

|  | *ex vivo PCa - PZ (midgland)* | | | *in vivo PCa - PZ (midgland)* | | |
| --- | --- | --- | --- | --- | --- | --- |
|  | **f** | **T2 [ms]** | **ADC [𝜇m^2^/ms]** | **f** | **T2 [ms]** | **ADC [𝜇m^2^/ms]** |
| **Epithelium** | 0.405 | 80.000 | 0.250 | 0.485 | 80.000 | 0.430 |
| **Stroma** | 0.350 | 50.000 | 0.800 | 0.355 | 50.000 | 1.480 |
| **Lumen** | 0.220 | 145.000 | 2.000 | 0.135 | 665.000 | 2.800 |
| **Vasculature** | 0.025 | 175.000 | 9.000 | 0.025 | 175.000 | 9.000 |


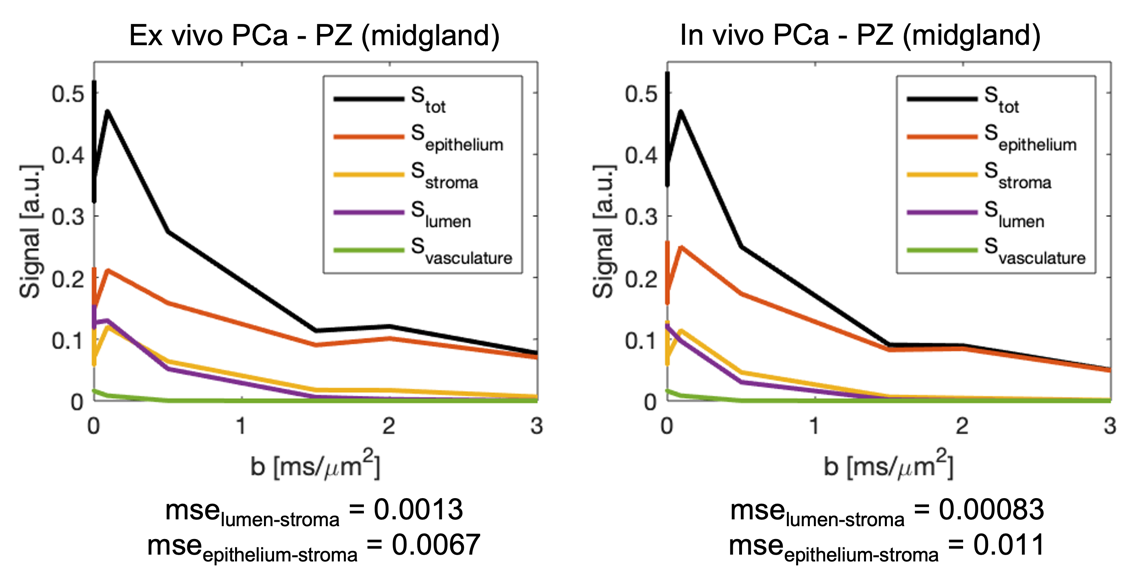


***Figure S3.*** *Comparison of the individual signal contributions to the total diffusion-weighted MRI signal (S_tot_) predicted according to the equation [S2] and the protocol used in this study. We focus on S_stroma_ and the assumption of classic VERDICT that its signal signature is more like S_lumen_ (lower mse) than S_epithelium_ (higher mse).*

We found that in both the ex vivo and in vivo simulated cases, the signal decay from stroma is closest to that of lumen (i.e., lowest mse), suggesting that, if only three compartments are modelled, then the lowest error can be achieved by coupling the signal from lumen and stroma, as the VERDICT model assumes.

Recent experimental evidence points towards stroma compartment having a T2 value closer to that of the epithelium^8,9^, we also assessed using these numerical simulations how assuming a unique average T2 for stroma and lumen could affect the accuracy of estimating f^0^_ic_. We have fitted rVERDICT to the S_tot_ we simulated for these two PCa conditions, adding Rician noise with SNR = 35 like in our experiments, and evaluated the average error of our estimates with respect to the ground-truth value of epithelial signal fraction over 1,000 different noisy instances. The results are reported in Supplementary Figure S3. We found that this assumption leads on average to underestimate the true epithelial signal fraction by ~14 percentage points in the ex vivo case and by ~20 percentage points in the in vivo case. This bias reduces to ~7 and ~10 percentage points, respectively, when high SNR = 100 can be achieved.

However, these results also show that in both cases, rVERDICT (red boxes in Figure S3) reduces the error of classic VERDICT (black boxes in Figure S3) on the estimated signal fractions modelling intracellular (fic), extracellular/extravascular (fees) and vascular (fvasc) compartments by respectively ~65, ~93 and ~12 percentage points for the ex vivo and ~64, ~83 and ~20 percentage points for the in vivo case.


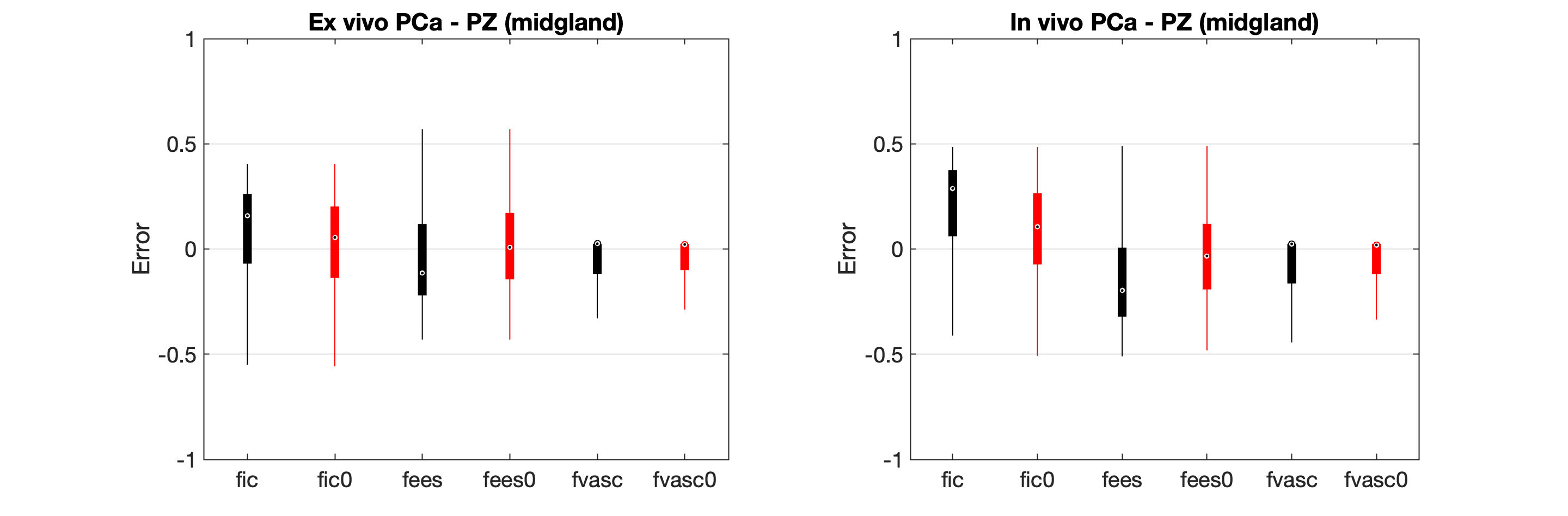


***Figure S4.*** *Comparison of the error (i.e. difference with the ground-truth values known by simulation design) in the estimated signal fractions modelling intracellular (ic), extracellular/extravascular (ees) and vascular (vasc) compartments using classic VERDICT (black boxes) and rVERDICT (red boxes), for ground-truth values representative of PCa in the PZ in both ex vivo and in vivo conditions, reported in the table above.*

**Accuracy of estimating the long T2 relaxation times**

As we have also highlighted in the limitation section in Discussion, the estimates of long T2 components may not be very accurate due to the limited maximal TE used in our sequence (90 ms). To assess how accurate our estimates of long T2 values are, we have performed simulations of a two-compartment system with a short (<=150 ms) and a long (>=150 ms) T2 component using equation [S1] and evaluated the accuracy and precision of the bi-exponential fitting using maximal TE of 90 ms and different SNR by adding corresponding Rician noise. We simulated 30x30x30 = 27,000 different combinations of short and long T2, with different signal fractions of short T2 component, obtained sampling a uniform grid with 30 steps: [signal fraction of short T2, short T2, long T2] = ndgrid(linspace(0,1,30), linspace(1,150,30), linspace(150,800, 30)). The results of these simulations are shown in Figure S4 and they suggest an error in the estimated values of the long T2 within ±5% of the true value for SNR = 35 (case of our experiments).


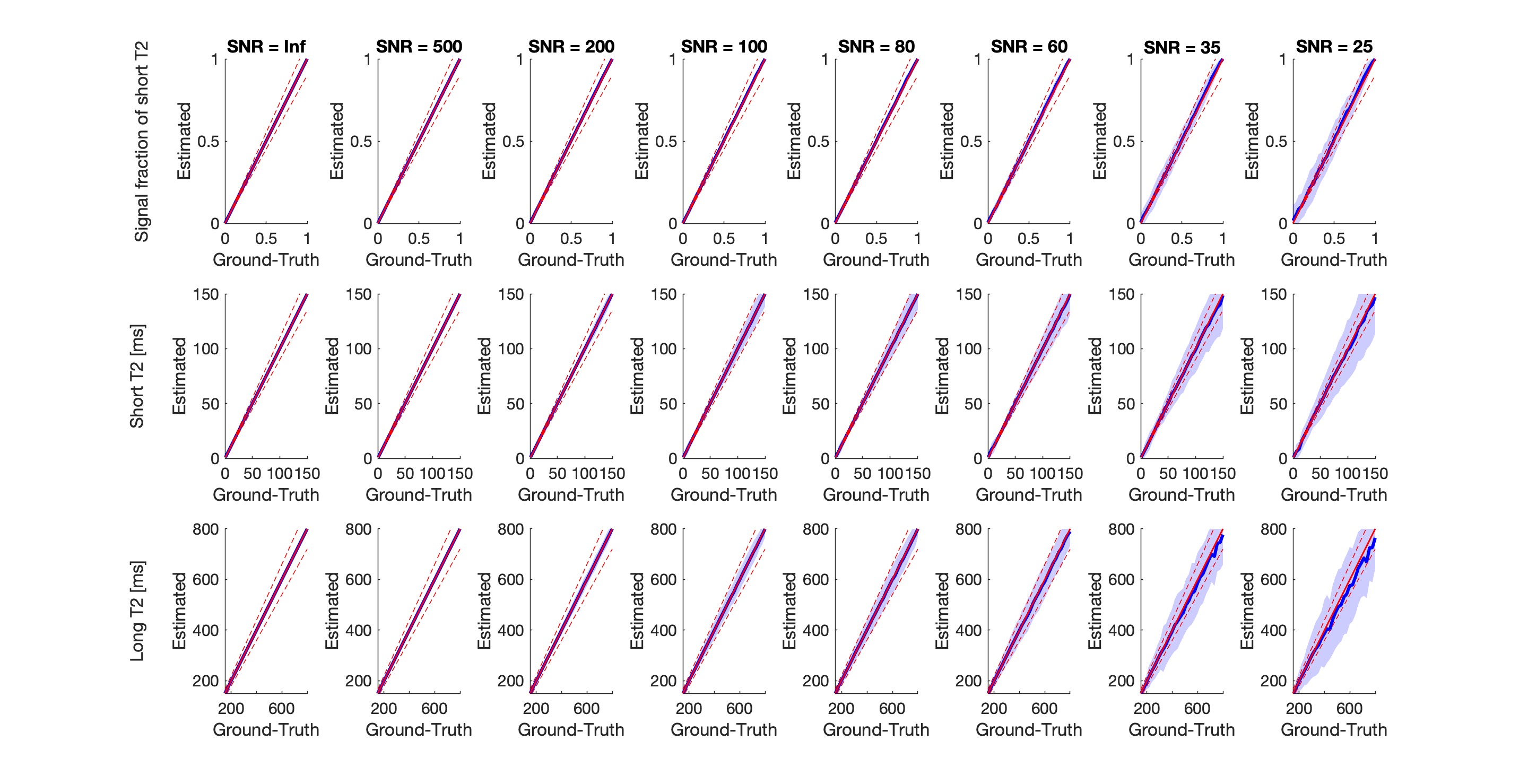


***Figure S5.*** *Accuracy and precision of the estimates of the two major T2 components in the prostate tissue using maximal TE of 90 ms, as in our experiments. The red solid lines show the identity lines; the red dashed lines the ±10% interval; the solid blue lines the mean estimates of the three parameters over different noisy instances; the blue shadows the corresponding standard deviation. We found that up to SNR ~ 60, we have very good accuracy for all the model parameters as well as small standard deviation (i.e. within ±10% of the true value), suggesting high precision. For progressively lower SNR values, the accuracy decreases, with underestimation of the long T2 components for T2 values >~ 400 ms. However, the error in the estimation of the long T2 component is within ±5% of the true value for SNR = 35 (case of our experiments).*

**Additional information on the regions of interest (ROI) definition**

The ROIs were informed by the biopsy locations determined by two reads of the participant's multiparametric MRI by uro-radiologists at our specialist centre. Differences in reports were resolved in a multi-disciplinary meeting. The biopsy targets were indicated on a pictorial report, which was used by a board-certified radiologist to draw ROIs on the lesions on the parameter maps. The radiologist was blinded to histology results. The pictorial reports were used by experienced urologists to carry out MRI-targeted biopsies using ultrasound guidance. MRI targets were matched to real-time ultrasound imaging using cognitive visual registration. This method has been used successfully at our centre for several years and results published in multicenter trials such as the PRECISION prostate trial ^11^. Unexpected biopsy results were also discussed at a multi-disciplinary meeting and repeated if there was a concern for sampling error. We did not include any false positives in this study. Most of the ROIs were 2D. Only in a few cases (less than 6 ROIs) the observable lesions extended to neighbouring slices and in those cases 3D ROIs were considered.

**Model’s parameters cross-correlation**

First, we investigated model’s parameters cross-correlations in the simulations (using noisy signals with SNR=35 similar to our experimental one) evaluating the Spearman’s rank correlation coefficient ρ between couples of model parameters. We found that no strong correlation exists between any of the estimated model parameters (see correlation matrix in Figure S6). This confirms that rVERDICT is not over-parametrized.

We then investigated pixel-wise correlations. The correlation matrix obtained considering all the voxels within the prostate of the three representative patients reported in Figure 7 is shown in Figure S6.

We found strong correlations (ρ>0.75) between:

- fic0 and fees0 (negative);
- fic0 and T1 (negative);

moderate correlations (0.50<ρ<0.75) between:

- fees and T1 (positive);
- fees0 and Dees (positive);
- T2vasc/ees and T2ic (positive);
- T2ic and T1 (positive);

and weak correlations (ρ<0.5) for the remaining couples of model parameters.

Finally, we performed similar correlation analysis at the patient level, using all the ROIs from all the patients. The resulting correlation matrix is shown in Figure S6.

We found strong correlation (ρ>0.75) only between fic0 and fees0 (negative);

moderate correlations (0.50<ρ<0.75) between:

- fic0 and T1 (negative);
- fic0 and T2ic (negative);
- fic0 and Dees (negative);
- R and T2ic (negative);
- fees0 and Dees (positive);
- T2vasc/ees and T2ic (positive);
- T2ic and T1 (positive);

and weak correlations (ρ<0.5) for the remaining pairs of model parameters.

**
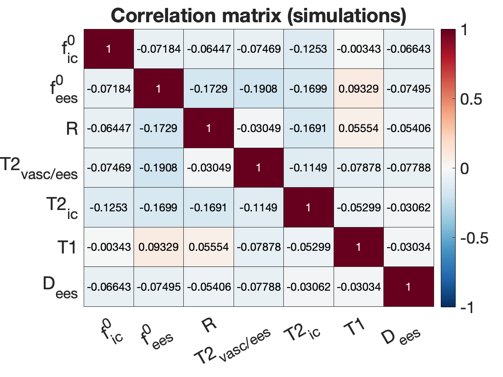

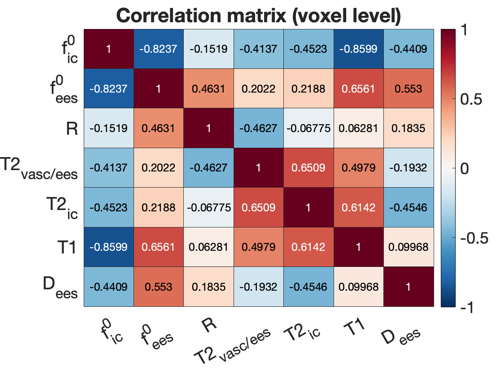

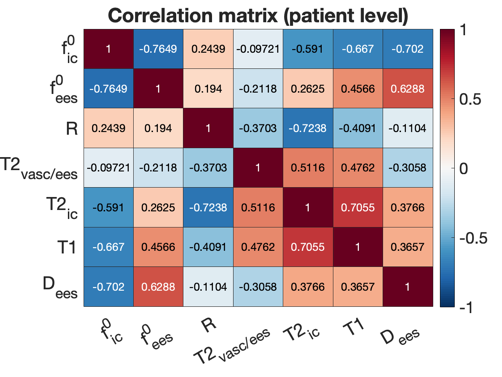
**

***Figure S6.*** *Model’s parameters cross-correlation analysis. The tree matrices show the Spearman’s rank correlation coefficient ρ (reported both in text and colour-coded according to the reported colour-map) between each couples of rVERDICT model parameters using three sets of data: simulated data (left) using noisy signals with SNR=35 similar to our experimental one; considering all the voxels within the prostate of the three representative patients reported in Figure 7 (centre) and using all the ROIs from all the patients (right).*

**References**

1 Scheenen, T. W., Rosenkrantz, A. B., Haider, M. A. & Futterer, J. J. Multiparametric Magnetic Resonance Imaging in Prostate Cancer Management: Current Status and Future Perspectives. *Invest Radiol* **50**, 594-600, doi:10.1097/RLI.0000000000000163 (2015).

2 Cook, P. A. *et al.* Camino: open-source diffusion-MRI reconstruction and processing. *In 14th scientific meeting of the international society for magnetic resonance in medicine (Vol. 2759, p. 2759). Seattle WA, USA.* (2006).

3 Kingma, D. P. & Ba, J. Adam: A method for stochastic optimization. *arXiv preprint arXiv:1412.6980* (2014).

4 Storas, T. H., Gjesdal, K. I., Gadmar, O. B., Geitung, J. T. & Klow, N. E. Prostate magnetic resonance imaging: multiexponential T2 decay in prostate tissue. *J Magn Reson Imaging* **28**, 1166-1172, doi:10.1002/jmri.21534 (2008).

5 Panagiotaki, E. *et al.* Microstructural Characterization of Normal and Malignant Human Prostate Tissue With Vascular, Extracellular, and Restricted Diffusion for Cytometry in Tumours Magnetic Resonance Imaging. *Invest Radiol* **50**, 218-227, doi:Doi 10.1097/Rli.0000000000000115 (2015).

6 Bailey, C. *et al.* VERDICT MRI validation in fresh and fixed prostate specimens using patient-specific moulds for histological and MR alignment. *Nmr Biomed* **32**, e4073, doi:10.1002/nbm.4073 (2019).

7 Bonet-Carne, E. *et al.* in *Proc. Intl. Soc. Mag. Reson. Med.*

8 Zhang, Z. *et al.* Prostate Microstructure in Prostate Cancer Using 3-T MRI with Diffusion-Relaxation Correlation Spectrum Imaging: Validation with Whole-Mount Digital Histopathology. *Radiology*, 192330, doi:10.1148/radiol.2020192330 (2020).

9 Chatterjee, A. *et al.* Diagnosis of Prostate Cancer with Noninvasive Estimation of Prostate Tissue Composition by Using Hybrid Multidimensional MR Imaging: A Feasibility Study. *Radiology* **287**, 864-873, doi:10.1148/radiol.2018171130 (2018).

10 Bonet-Carne, E. *et al.* VERDICT-AMICO: Ultrafast fitting algorithm for non-invasive prostate microstructure characterization. *Nmr Biomed* **32**, doi:ARTN e4019

10.1002/nbm.4019 (2019).

11 Kasivisvanathan, V. *et al.* MRI-Targeted or Standard Biopsy for Prostate-Cancer Diagnosis. *N Engl J Med* **378**, 1767-1777, doi:10.1056/NEJMoa1801993 (2018).
